# COVID-19 in conflict region: the arab levant response

**DOI:** 10.1186/s12889-021-11580-4

**Published:** 2021-08-26

**Authors:** Nazih A. Bizri, Walid Alam, Tala Mobayed, Hani Tamim, Maha Makki, Umayya Mushrrafieh

**Affiliations:** 1 Faculty of Medicine, University of Balamand, Koura, Lebanon; 2 Department of Internal Medicine, American University of Beirut Medical Center, Beirut, Lebanon; 3 Clinical Research Institute, Biostatistics Unit, American University of Beirut, Beirut, Lebanon; 4 Department of Family Medicine, American University of Beirut Medical Center, Beirut, COVID-19 Unit Director, American University of Beirut Medical Center, Beirut, Lebanon

**Keywords:** COVID-19, Arab Levant, War, Conflict, political stability index, world governance indicators, Lebanon, Iraq, Jordan, Palestine, Syria, political climate

## Abstract

**Background:**

COVID-19 has hit the world in an unprecedented way causing serious repercussions on several aspects of our life. Multiple determinants have affected various nations’ level of success in their responses towards the pandemic. The Arab Levant region in the Middle East, notoriously known for repeated wars and conflicts, has been affected, similarly to other regions, by this pandemic. The combination of war, conflict, and a pandemic brings too much of a burden for any nation to handle.

**Methods:**

A descriptive analysis of data obtained from the health departments of various Arab Levant Countries (ALC) was performed. ALC include Lebanon, Syria, Jordan, Iraq and Palestine. The data collected involves incidence, recovery rate, case fatality rate and number of tests performed per million population, Global Health Security index, government stringency index, and political stability index. The information obtained was compared and analyzed among the ALC and compared to global figures. An extensive electronic literature search to review all relevant articles and reports published from the region was conducted. The 2019 Global Health Security (GHS) index was obtained from the “GHS index” website which was made by John Hopkins University’s center for health security, the Nuclear threat Initiative (NTI) and the Economist Intelligence Unit (EIU). Government stringency index and political stability index were obtained from the University of Oxford and the website of “The Global Economy”, respectively. Other world governance indicators such as government effectiveness were obtained from the World Bank website.

**Results:**

In terms of incidence of COVID-19, Iraq has the highest with 9665 per one million population, Syria the lowest at 256 per million. Deaths per million population was highest in Iraq at 237, and the lowest in Syria at 12. As for number of tests per million population, Lebanon ranked first at 136,033 with Iraq fourth at 59,795. There is no data available for the tests administered in Syria and subsequently no value for tests per million population. In terms of recoveries from COVID-19 per million population, Iraq had the highest number at 7903 per million, and Syria the lowest at 68 per million. When compared as percent recovery per million, Palestine ranked first (84%) and Syria last (27%). The government response stringency index shows that Jordan had the highest index (100) early in the pandemic among the other countries. Palestine’s index remained stable between 80 and 96. The other countries’ indices ranged from 50 to 85, with Lebanon seeing a drop to 24 in mid-August. Even with improved stringency index, Iraq reported an increased number of deaths.

**Conclusion:**

In countries devastated by war and conflict, a pandemic such as COVID-19 can easily spread. The Arab Levant countries represent a breeding ground for pandemics given their unstable political and economic climate that has undoubtedly affected their healthcare systems. In the era of COVID-19, looking at healthcare systems as well as political determinants is needed to assess a country’s readiness towards the pandemic. The unrest in Lebanon, the uprising in Iraq, the restrictions placed on Syria, and the economic difficulties in Palestine are all examples of determinants affecting pandemic management. Jordan, on the contrary, is a good example of a stable state, able to implement proper measures. Political stability index should be used as a predictor for pandemic management capacity, and individual measures should be tailored towards countries depending on their index.

## Background

COVID-19 is a global pandemic which has caused disruption in various aspects of our daily life. When faced with this pandemic, each country responded differently [1, 2]. The virus has taken the world by surprise and, in just a few months, the number of cases worldwide were in the millions and the number of deaths in the hundreds of thousands. Many countries have had different success in dealing with the virus. However, one region, the Levant, is of significance since many of its countries have recently witnessed violence, civil wars, uprisings and political unrest. Public distrust in the ruling elite is a common factor shared by most ALC and is problematic for many in the public health care system, as it reflects distrust towards the capacity of governments to provide accurate data about the extent of the pandemic [3].

COVID-19 is a data driven disease, where everyone is constantly learning and adapting based on the new information and studies published about the disease daily. This is crucial since a country’s response to the pandemic depends on how well established and functional its health care systems are [4]. The degree of preparedness to face a new health challenge and the quality of the health response in a country is reflected by disease reporting, surveillance, diagnostic testing, mortality and recovery rates. As per the World Health Organization (WHO), countries with a low-quality health infrastructure will ultimately suffer from slower improvement in community health and increased burden of disease [5].

The Levant region which includes Palestine, Lebanon, Syria, Iraq and Jordan [6] is a peculiar one. These countries are conflict-laden and encompass politically fragmented areas, putting them at a disadvantage when initiating response and managing present or emerging diseases in their countries [7]. The aim of this article is to assess and compare ALC response to the COVID - 19 pandemic in relation to several variables that include: number of cases per million, number of deaths per million, recoveries per million and number of diagnostic tests performed per million, global health safety index, government stringency index, political stability index, and world governance indices. This is essential to reveal and highlight the challenges these countries faced during the pandemic, and to determine why some countries of the Levant region with more corruption and less stability had a less effective health system response than others countries of that region with less corruption and better stability. All of this will aid in better understanding the pandemic, facilitating better control of COVID-19 in an attempt to maximize correct health management once the pandemic is over.

## Methods

Cumulative data pertaining to incidence, death, recoveries and testing were obtained from the website of the Ministry of Health of each country up to October 16, 2020. An extensive literature review using the following search engines: PubMed, Medline and Google Scholar was conducted using the key words “COVID-19′, “Palestine”, “Lebanon”, “Syria”, “Iraq”, and “Jordan”. Relevant data from websites belonging to WHO, Relief Web, Organization for Economic Co-operation and Development (OECD) and respective Ministries of Health were obtained. Rates per 1 million population were used for standardization to compensate for the discrepancy in population sizes and for rankings. Global Health Safety (GHS) index, October 2019 was obtained from the “GHS index” website which was made by John Hopkins University’s center for health security, the Nuclear threat Initiative (NTI) and the Economist Intelligence Unit (EIU). Government stringency index and political stability index were obtained from the University of Oxford and the website of “The Global Economy”, respectively [8, 9]. Other world governance indicators such as government effectiveness and regulatory quality were obtained from the World Bank website [10].

The indices were chosen based on their credibility and trusted resources that include up to date and accurate information. They stand as an essential tool to study and compare countries in a broad sense, while highlighting improvement and trends of a particular country. The data is ongoing and susceptible to change. Furthermore, the government stringency index was retrieved from the Oxford Covid-19 Government Response Tracker (OXCGRT) which systematically collects data from diverse governments’ reactions, and scores the stringency of such measures into a common stringency index. All these indices target precisely the paper’s aim to investigate the efficacy of the different government responses to the pandemic, midst political and economic crisis. Most importantly, there were selected based on their wholesome data that include countries globally, including Palestine since it was challenging to find information about Gaza, Palestine, a country of major conflict.All data were analyzed and summarized using descriptive statistics, with the results displayed in tables and discussed in relevance to the literature search. Discussion was made and derived for each country alone. A *p*-value < 0.05 was considered statistically significant.

### Definitions [8, 10–12]

#### Confirmed Positive Case

A confirmed case is where the reverse transcription polymerase chain reaction (RT-PCR) tests are positive regardless of presenting symptoms.

#### Confirmed Negative Case`

A negative case is where two consecutive RT-PCR tests are negative.

#### Coronavirus Government Stringency Index

A composite measure of nine metrics which are school closures; workplace closures; cancellation of public events; restrictions on public gatherings; closures of public transport; stay-at-home requirements; public information campaigns; restrictions on internal movements; and international travel controls. This measured is rescaled to a value from 0 to 100 (100 = strictest).

#### Political Stability Index

The index of Political Stability measures perceptions of the likelihood that the government will be destabilized or overthrown by unconstitutional or violent means, including politically motivated violence and terrorism.

#### Regulatory quality

captures perceptions of the ability of the government to formulate and implement sound policies and regulations that permit and promote private sector development.

The index of *Government Effectiveness* captures perceptions of the quality of public services, the quality of the civil service and the degree of its independence from political pressures, the quality of policy formulation and implementation, and the credibility of the government’s commitment to such policies.

#### The Global Health Security Index

Assess country needs and capacity to prevent, detect or control a future biological threat, creating better comprehension of proven global capabilities in responding to a pandemic. It analyzes the health security across 195 countries and is organized across 6 categories: Prevention (preventing the emergence or release of pathogens) - Detection and Reporting Epidemics of Potential International Concern- Rapid Response-Health System- Health (Robust health sector to treat the sick and protect health workers) - Compliance with International Norms- Risk Environment to biological threats. The scoring system is based on a scale of 0–100 index score (index score 100 = best health security conditions).

## Results

**Fig. 1:**
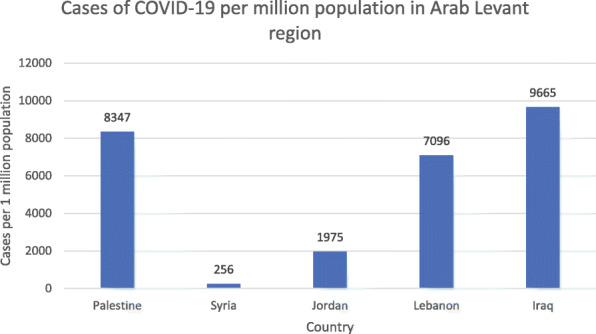
Cases of COVID-19 per million population in Arab Levant region

**Fig. 2:**
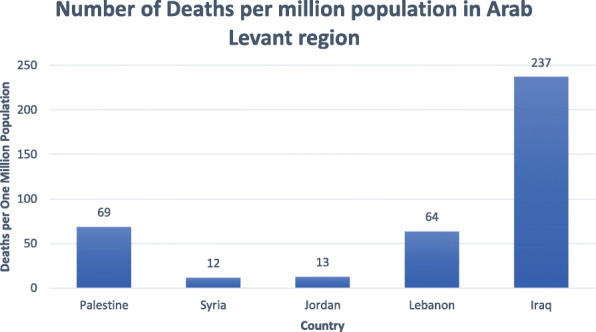
Number of deaths due to COVID19 per million population in Arab Levant region

**Fig. 3:**
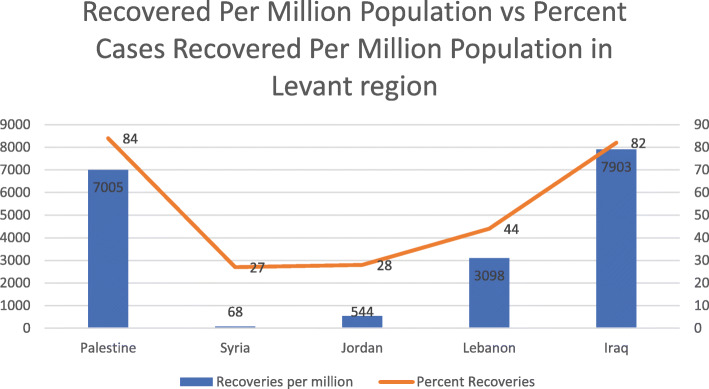
Number of recoveries per million population and percent cases recovered per million population in Arab Levant region

**Fig. 4:**
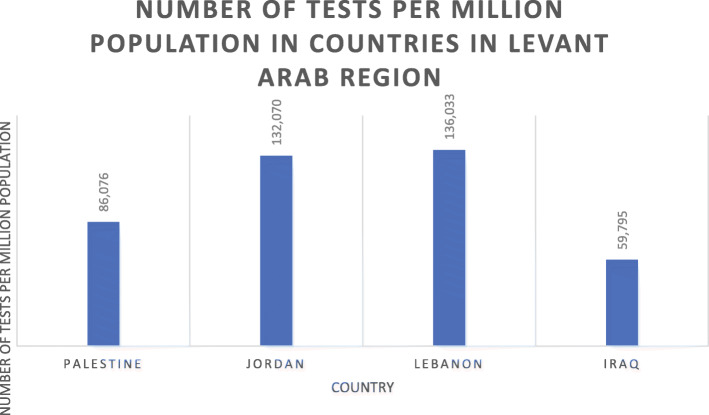
Number of tests per million population in countries in Levant Arab region

All data mentioned is per 1 million population and is summarized in the tables below [13–15]. In terms of incidence of COVID-19, Iraq ranks first (9665), followed by Palestine (8347), then Lebanon in third place (7096), Jordan in fourth (1975), and lastly Syria (256) (*P* < 0.0001) (Table 1, Fig. 1). With respect to mortality, Iraq ranks first in deaths per million population at 237 and Syria last at 12 (Table 1, Fig. 2). As for number of tests per million population, Lebanon and Jordan ranked first (136,033) and second (132,070), respectively. There is no data available for the tests administered in Syria and subsequently no value for tests per million population (*P* < 0.0001) (Table 2, Fig. 3). In terms of recoveries per million population, Iraq ranked first (7903), and Syria last (68). When compared as percent recovery per million, Palestine ranked first (84%), and Syria last (27%) (*P* < 0.0001) (Table 3, Fig. 4).

**Table 1:** Total number of COVID cases, deaths and recovery in the Arab Levant countries as of October 16, 2020

Country	Population	Total Number of Cases	Total Deaths	Total Recovered	Total Tests
**Palestine**	5,132,482	42,840	355	35,953	441,785
**Syria**	17,611,826	4504	212	1198	N/A
**Jordan**	10,230,283	20,200	131	5575	1,351,112
**Lebanon**	6,817,067	48,377	433	21,120	927,346
**Iraq**	40,459,293	391,044	9604	319,784	2,419,252

**Table 2:** Comparison of COVID-19 cases, deaths, and recovery as expressed per one million of the population as of October 16, 2020 in Arab Levant countries

Country	Total cases/ million Population	Deaths / million Population	Recovered / million Population	Tests/ million Population
**Palestine**	8347	69	7005	86,076
**Syria**	256	12	68	N/A
**Jordan**	1975	13	544	132,070
**Lebanon**	7096	64	3098	136,033
**Iraq**	9665	237	7903	59,795

**Table 3:** Percent Recovery per million population from total cases per million population in Arab Levant countries as of October 16, 2020

Country	Percent Recovered (%)
**Palestine**	84
**Syria**	27
**Jordan**	28
**Lebanon**	44
**Iraq**	82

**Fig. 5:**
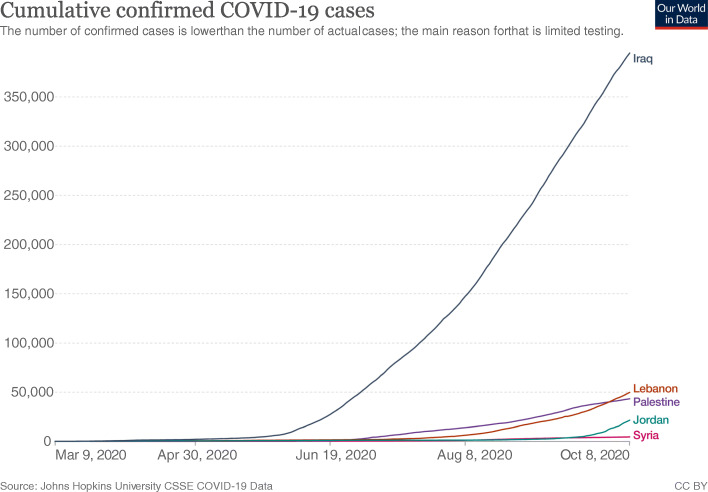
Cumulative COVID-19 cases among Arab Levant Countries. Data from https://ourworldindata.org/covid-cases?country=LBN~PSE~SYR~JOR~IRQ

From March to early October, Iraq had by far the highest cumulative confirmed COVID-19 cases among ALCs. Palestine and Lebanon had almost the same level of increase, followed by Jordan and finally Syria with the lowest cumulative cases (Fig. 5).

**Fig. 6:**
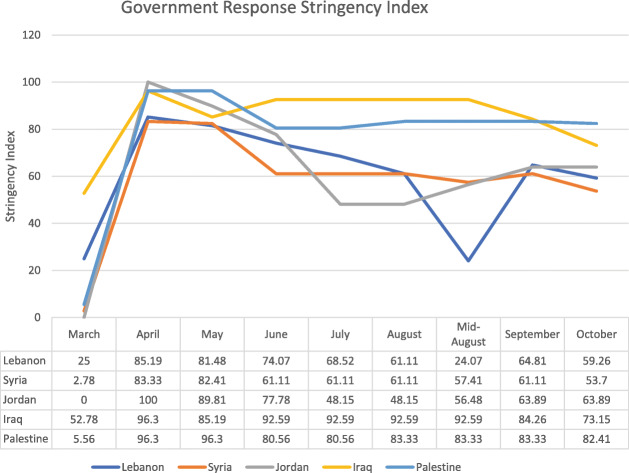
COVID-19: Government Response Stringency Index. Data collected from the University of Oxford

**Fig. 7:**
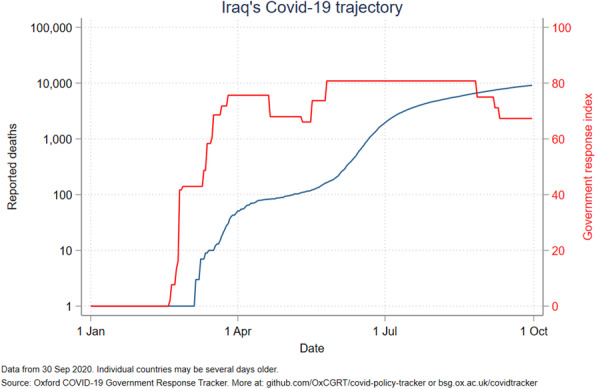
Iraq’s COVID-19 reported deaths and government response index from January to October 2020

**Fig. 8:**
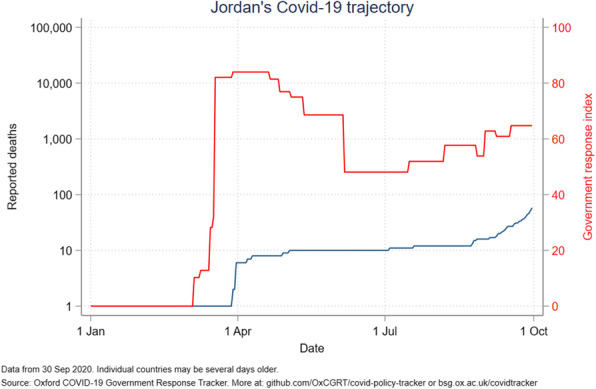
Jordan’s COVID-19 reported deaths and government response index from January to October 2020

**Fig. 9:**
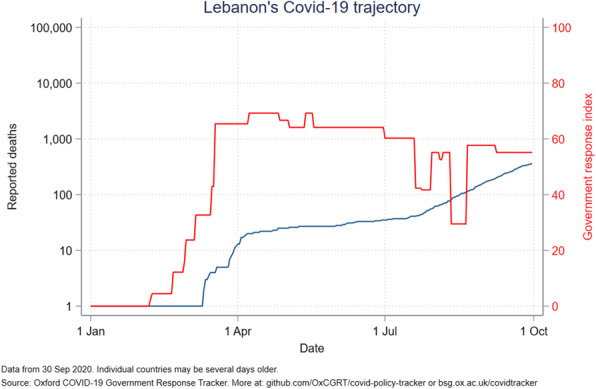
Lebanon’s COVID-19 reported deaths and government response index from January to October 2020

**Fig. 10:**
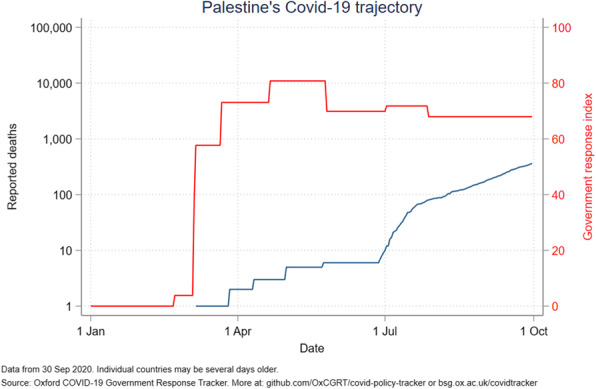
Palestine’s COVID-19 reported deaths and government response index from January to October 2020

**Fig. 11:**
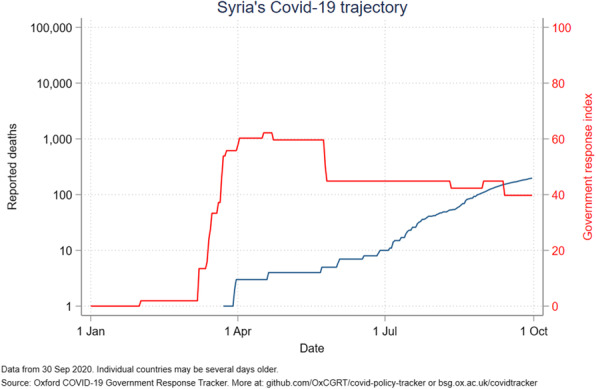
Syria’s COVID-19 reported deaths and government response index from January to October 2020

The government response stringency index shows that Jordan had the highest index (100) early in the pandemic, subsequently decreasing to 58 in July and August before increasing to 63 in October. Palestine had the most stable index from April to October, ranging from 80 to 96. The other countries’ indices ranged from 50 to 85, with Lebanon seeing a drop to 24 in mid-August (Fig. 6). Even with increased measures, Iraq reported an increased number of deaths (Fig. 7). A direct relation was observed between reported deaths and government response stringency index in the remaining countries (Figs. 8, 9, 10 and 11).

**Fig. 12:**
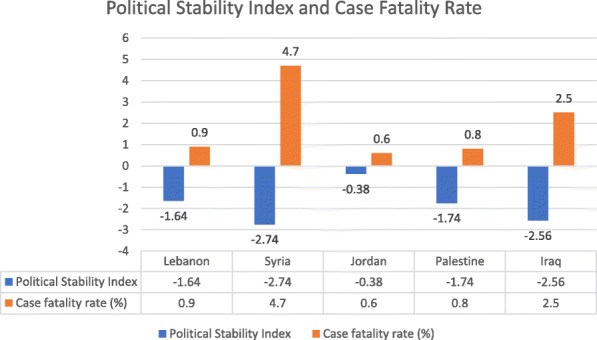
Political Stability Index and COVID-19 Case Fatality Rate Among Arab Levant Countries. Data retrieved from the Global Economy website https://www.theglobaleconomy.com/rankings/wb_political_stability/

**Fig. 13:**
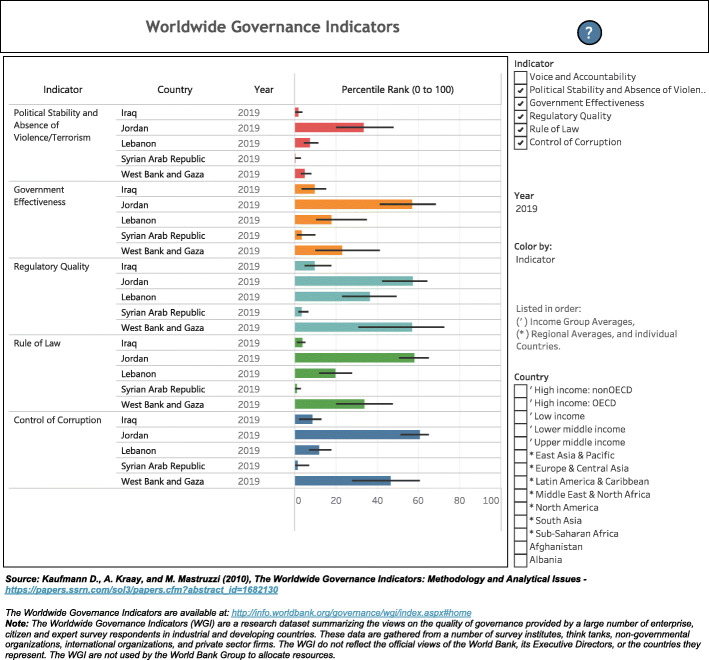
*World Governance Indicators of Arab Levant Countries. Data obtained from WorldBank,*https://info.worldbank.org/governance/wgi/Home/Reports*.* *West Bank and Gaza refer to Palestine

Among the ALCs, Jordan had the best political stability index (− 0.34) with Syria the worst (− 2.76). Similarly, COVID-19 case fatality rate was the lowest in Jordan (0.6) and the highest in Syria (4.7). Palestine and Lebanon are close in case fatality rate and political stability index, while Iraq is second to Syria (Fig. 12). The world governance indicators of ALCs shows again that Jordan is the best, followed by Lebanon, Palestine (West Bank and Gaza), Iraq, and lastly Syria (Fig. 13).

Syria has the lowest Global Health Security Index score (19.9) among the ALCS, being ranked as 188/195 countries in terms of health safety. On the other hand, Lebanon ranked as 73/195 countries, having the best average index score (43.1) among the 5 mentioned countries. The Global Health Security Index again shows that Syria is the worst, followed by Iraq (25.8 index score, 167/195), but this time with Lebanon having the best index score instead of Jordan (42.1 index score,80/195) (Table 4).

**Table 4:** Comparison of the 2019 Global Health Security Index between each of the Levant Countries. Data for Palestine is unavailable

	Prevention Index score	Detection & Reporting Index score	Rapid Response Index score	Health System	Compliance with International Norms Index score	Risk Environment Index score	Overall Average Index Score	Rank
Syria	18.4	2.7	23.0	24.4	26.1	29.6	**19.9**	**188/195**
Iraq	22.1	42.2	19.5	11.8	29.5	29.2	**25.8**	**167/195**
Jordan	31.8	42.9	47.8	27.8	48.6	55.8	**42.1**	**80/195**
Lebanon	27.3	62.0	47.9	23.8	49.3	45.5	**43.1**	**73/195**

## Discussion

### Syria

Syria is a country that is currently entering its ninth year of conflict. Prior to the conflict, Syria’s health system was comparable with that of other middle-income countries [16]. However, the prolonged struggle has led to significant destruction and shattering of the health infrastructure. The health infrastructure was suboptimal, particularly outside major cities, with insufficient facilities and inadequate equipment [17]. Besides the fractured healthcare system due to conflicts, the system is being further destroyed by sanctions [18].

Concerning the battle against COVID-19, great efforts by the WHO and the Syrian Ministry of Health were made to trace, track, and isolate COVID-19, but the balkanized nature of the country made success difficult [19]. When compared to the other countries in the region, Syria suffers from lack of data, and the least number of recoveries. There were several contributing factors like lack of organization and preparedness for the pandemic [20]. In addition, the conflict has led to the departure of more than 70% of the healthcare work force and death of physicians with no opportunity to train a new generation of healthcare workers. Also, the number of doctors left in Syria who are qualified to deal with COVID-19 patients is quite limited. The healthcare system was operational at around only 50% of its capacity [21]. Even though the number of cases is the lowest among those in other countries in the region, the Syrian Ministry of Health has a history of failing to report communicable diseases as was the case in 2013 with the rise of Polio cases, a failure attributed to the political influences of the conflict. Furthermore, the surveillance systems are poor, with unstable conditions in many regions and no standardized method for reporting infections. Justifiable concern exists in that the real figures are much higher, and that a winter surge will occur unless the virus is contained now [22, 23]. The lack of testing only serves to increase the concern over the actual number of cases, especially that neighboring countries seemingly reported confirmed cases of COVID-19 long before Syria declared their first infection [7]. Syria suffers from several gaps in its sanitary infrastructure which include inadequate supply of Personal Protective Equipment (PPE), poor efforts to prevent cross infections within and out of health facilities, inadequate laboratory and case investigations, and deficient efforts in safeguarding of public health [24]. There is also an US$11 million funding gap until the end of the year for the COVID-19 preparedness and response efforts, a gap that is vital to fill for testing expansion, surveillance strengthening, and furthering IPC materials [25].

When looking at Syria’s political stability index, it is the lowest among ALCs and has seen a sharp decline since the start of the Syria crisis in 2011, reaching a classification of weak and the second lowest index among countries worldwide in 2018 [26]. Other world governance indicators such as government effectiveness, and regulatory quality are also the lowest among ALCs **(**Fig. 13**)**. Moreover, the government response index to the pandemic has been borderline low since June **(**Fig. 11**)**. The inadequate efforts by the government explains the continuous rise of cases and reported deaths in Syria. However, while Syria shows the lowest number of cumulative cases, the numbers massively underrepresent the reality of the situation as barely any testing is done and data concerning the number of tests per million population is not available **(**Figs. 4 and 5**)**. As such, the Detection and reporting index of the Global Health Security Index was extremely low (2.7) **(**Table 4**)**. In fact, conflict laden countries testing is reliant on resource-limited settings and requires support from other entities such as the WHO. Timely transport of samples to laboratories is also harder with poor road infrastructure, and preservation of these samples and the reagents is made impossible with frequent power outages [27, 28]. All these obstacles are evident by the indices used which could help predict why the case fatality rate in Syria is the highest, and why control and management of a pandemic is challenging for that country.

Despite the billions of euros donated by the European union (EU) to aid Syria and adjacent countries, and directed mostly towards refugees and the displaced, the combined effect of the EU countermeasures and the US Caesar Act produced serious isolation and enduring harm to the Syrian people and is currently preventing the Syrian Government from fully addressing the pandemic [19].

### Iraq

Iraq suffered through corruption, sectarian tensions, a civil war, political turmoil, and an extremist insurgency leaving it politically and economically vulnerable [29]. Iraq is ranked first in the number of cases, deaths, and recoveries. In response to the coronavirus disease (COVID-19) pandemic, Iraqi authorities have imposed mobility restrictions since March 2020 aimed at curbing the spread of the virus. These measures included restrictions on travel and limitations on freedom of movement, such as the closure of airports and points of entry along land borders and maritime boundaries, as well as domestic movement restrictions [30]. In June, there was a 600% rise in COVID-19 cases in Iraq and there was a call from the International Rescue Committee to re-double efforts to slow the spread of the disease [31]. The increase in cases is due to several factors, namely the relaxation of restrictions, and the sharp increase in the number of patients that were noticed with an increase in case fatality. Despite this increase, there was a perceived unwillingness of the society to follow the instructions of social distancing and infection prevention. Mistrust in the government and myths that coronavirus was political game became widely held beliefs. Add to that, the fragile weakened socioeconomic state impacted the lockdown due to the reduction of community cohesion, loss of education, widespread loss of jobs and insecurity of food. This led to a breakdown in relations between the society and local authority and therefore the community was unwillingly fighting the pandemic [32]. Other contributing factors were the protests that continue to occur across the country, and the commemoration of pilgrims, all defying the restrictions and the required social distancing.

Baghdad is a populated capital with around ten million citizens and many expatriates coming from their travel from banned countries [33]. It is worth noting that the persistent conflict led to the displacement of the citizens of Iraq, and a collapse of immunity and health program of communicable diseases as was the case with measles reporting [34]. This turbulent ambience of conflict on one hand, and the fractured surveillance system on the other hand, resulted in a low number of tested people. Therefore, an ever-greater number of cases most likely exists, a number unlikely to be properly detected with the Central Public Health Library only performing 100 screening tests per day as of April 30, 2020. But, with the opening of a new molecular lab donated by the People’s Republic of China, and the availability of improved tools to identify positive cases, the number of tests, and consequently cases, should increase [33].

When looking at the political index and Global health Security Index, Iraq has one of the worst among ALCs, second only to Syria **(**Fig. 12**) (**Table 4**)** and ranking fifth in the world [35]. The political index also correlates with the country’s second highest case fatality rate, and its highest cumulative COVID-19 cases which is exponentially higher than the rest of the ALCs **(**Fig. 5**)**. The government’s response has not been stable, and its stringency index has recently dipped, with an associated increase in reported deaths **(**Fig. 7**)**. Indicators for government effectiveness, and regulatory quality in Iraq are only mildly higher than that of Syria’s **(**Fig. 13**)**. These indicators help explain the distrust of citizens towards their government and their unwillingness to follow measures, but also how inadequate restrictions were implemented and are contributing to a continuous increase in case numbers.

### Palestine

Palestine has been subjected to conflict for decades. Political and military siege plague the area, and have led to political, economic, and social instabilities [36]. The pandemic added further insult to a country already suffering from occupation and intra-country divide. The high number of cases could be explained by several factors such as overcrowding and unsanitary conditions in camps, poor humanitarian needs, and refugees [37]. Although Palestine followed a set of global standards and procedures to tackle the pandemic, they had to maneuver within several constraints: weak health infrastructure, a fragile economy, and an unstable political climate [38]. Despite the strict measures taken early by Palestine to contain the situation and prevent rapid spread of the virus in the country, the real challenge started when the Palestinian workers in Israel returned home after the spread of COVID-19 there. The total number of workers amounted to more than 45,000 individuals, making it challenging for Palestine to implement proper accommodation, testing, and quarantine in suitable locations. Authorities feared a widespread of COVID-19, given the Palestinian Ministry of Health’s inability to deal with hundreds of cases due to the lack of necessary capacities in the available hospitals, threatening a total collapse of the health system in Palestine [39]. The country responded by enforcing social distancing measures, lockdown of religious sites and suspending all sorts of educational activities. Nonetheless, the poor humanitarian situation put the country’s capacity to mitigate the effects of the virus at a disadvantage. Lack of sanitation and hygienic water sources facilitated the spread of communicable diseases. Low funding for healthcare systems has led to a deficiency in adequate screening and lack of availability of proper protective equipment [40].

While the number of tests per million is the 3rd highest among ALCs, more tests need to be made accessible by the Palestinian government (Fig. 4). Acute shortages of laboratory supplies and equipment needed for COVID-19 testing remain a significant challenge, with the Gaza central laboratory projecting 200,000 tests being needed until end December 2020. Healthcare services are still affected by critical shortages of essential drugs and disposables. As of end of September 2020, 47% of essential drugs were at zero stock level (less than 1 month’s supply), leading to inadequate medical care for the most vulnerable patients. 50% of primary healthcare staff are re-assigned to support the COVID-19 response, leading to a compromise in primary healthcare service. As with other countries, there are important shortages of PPEs used by frontline health workers. 150,000 full PPE kits, 1 million surgical masks and 2 million gloves, are needed for the response over the next three months [41].

Palestine has decreased its government stringency index in June which has led to an increase of reported deaths and cumulative cases **(**Figs. 5 and 10**)**. Movement restrictions are being eased, and local markets are being partially reopened. A curfew from 20:00 to 07:00 remains in place in all governorates. Schools will be reopened on October 10, with around 35,000 students expected to return as an initial step which would drastically reduce the stringency index [41].

When compared to all ALCs, Palestine ranks third in political stability index and other world governance indicators **(**Figs. 12 and 13**)**. Case fatality rate is also low, second to Jordan **(**Fig. 12**)**. The relative political stability and government trust when compared to Iraq and Syria has allowed Palestine to manage the pandemic significantly better, with restrictions implemented and respected by the citizens, and testing done to full possible capacity of the country. Palestine’s ability to control spread is largely due to the area being contained between Jordan and Israel, with lack of aerial travel to or from the country, limiting the import of cases [42]. However, the indices reflect Palestine’s inability to accommodate testing for all its population and to provide enough PPEs, and the inadequate number of healthcare workers needed to both manage the pandemic while also delivering proper general healthcare services.

### Lebanon

Facing years of political corruption causing societal divide, and economic inequality, Lebanon finally reached a breaking point in its economy, and now faces a recession and near total collapse. The situation worsened when an unexpected explosion took place near the country’s main capital leaving millions in damages, and thousands of lives affected [43]. Lebanon is an example of a country where the initial response to COVID-19 was exemplary. However, the lack of infrastructure, resources and adequate funds have left many hospitals without enough personal protective equipment and hospital beds, to meet the growing number of cases. In comparison to the other countries in the Levant region, Lebanon ranks third in cases, deaths and number of people recovered. These low numbers can be attributed to several factors such as a young age distribution, strict policies regarding travel, closure of all educational institutions starting on 29 February, the national curfew by the Lebanese government, and a lack of public spaces and public transportation. With respect to testing, Lebanon suffered a lack of funds and resources, and scarcity in foreign currency that have positioned the country at a critical spot in facing the COVID-19 pandemic. This depletion of resources needed to import good quality diagnostic kits was a critical factor [44]. The Lebanese Ministry of Public Health originally assigned Rafik Hariri University Hospital as the sole center to conduct PCR tests which led to a logjam of tests that were not performed [43]. Lebanon took a more aggressive approach with social distancing measures and early lockdown that was deemed to be a necessity in response to the scarcity of resource for both their screening and treatment [45].

The massive dip of the government response index to 24 in mid-August caused a massive increase in cases and reported deaths **(**Fig. 9**)**. This decrease in stringency is largely due to the Beirut port explosion that occurred on August 4th, 2020 and left hundreds of thousands injured and homeless, with several infrastructures destroyed [46]. The government remained relatively lax in its restrictions with a borderline above average index in October while daily cases reached over 1000. Lebanon’s vulnerable economy has made it hard for the government to implement adequate restrictions. Lebanon’s political stability index is second to Jordan among ALCs but does not take into account the events of this year with the economic collapse, government dissolution, and general instability **(**Fig. 12**)**. As such, other world governance indicators have likely worsened but should remain above Palestine, Syria, and Iraq **(**Fig. 13**)**. Lebanon’s control problem relates directly to its economic depression and the Beirut port explosion which affected compliance in restrictions, and trust in the government. Alternatively, Lebanon has the 3rd lowest case fatality rate among ALCs at, slightly above Palestine’s rate **(**Fig. 12**)**. Predictably, Lebanon’s cumulative cases have been increasing and are still below that of Palestine and Iraq **(**Fig. 5**)**. Yet, the Global Health Security index gave Lebanon a high detecting and reporting index score making it exceed Jordan’s overall index score and ranking **(**Table 4**).**

### Jordan

While Jordan faces political and economic challenges, it is considered an anchor of stability in a region shaken by crises [47]. Jordan ranks fourth among the Levant countries and recoveries, fifth in deaths, and first in testing in the Levant region. Jordan’s unique handling of the coronavirus pandemic lies less in the specific measures imposed, but more so in the swift and aggressive fashion by which they were carried out. Jordan’s government implemented several awareness campaigns to ensure social distancing measures, with renewing calls to adhere to necessary safety measures.

First, social media was used to inform the population about the dangers of the virus and the need for social distancing. Strict lockdown measures were enforced by daily street patrols, mandatory curfews, and the compulsory closure of businesses and restaurants across Jordan. The government also offered citizens doorstep delivery of essential goods and sent truckloads of subsidized bread to distribute throughout different Amman municipalities [48]. Second, at risk groups such as children and elderly were under strict restrictions to ensure their safety. Third, religious and public figures were recruited to help spread awareness as these are the people that the population mostly listens to [48].

Additionally, since the start of the pandemic, the Government of Jordan has included refugees in the National Health Response Plan and gave them access to national health services similarly to Jordanian nationals, including referral of suspect cases to quarantine sites and requisite treatment. Refugee camps were also put under restrictions of movement since March, with only essential staff given access.

All these measures were aided by the public’s trust in the governmental approach, which was perceived positively, with Jordanians considering the government to have been successful in controlling the pandemic. Moreover, the government undertook several steps to ensure that the health sector was fully equipped to deal with the situation, such as increasing health systems capacity to take new cases, purchasing equipment and supplies, and employing a concentrated effort on tracking and tracing emerging cases. All of the above with the goal to flatten the curve, a goal successfully established [49].

Jordan has the best political index among ALCs and exceeds them considerably at four times the index of Lebanon who is 2nd to Jordan. Its world governance indicators, especially government effectiveness, rank first among those of ALCs **(**Figs. 12 and 13**)**. Jordan has the lowest case fatality rate at 0.6%, in line with its ranking in political stability. Importantly, Jordan implemented necessary measures to earn a maximal government stringency index in April, which led to a massive decrease in its cases **(**Fig. 6**)**. As such, Jordan received the highest prevention index score, the highest health system index score, and the highest risk environment index score in the Global Health Security Index **(**Table 4**).** Restrictions were eased but are now being reinforced due to a re-increase in cases and reported deaths **(**Fig. 8**)**. Jordan’s political stability has allowed it to not only fully implement necessary restrictions, but to also provide adequate testing and care for both its citizens and refugees.

## Conclusion

COVID-19 infects the most vulnerable populations, and the current pandemic is now attacking the global health system, with areas of war and conflict seemingly the best targets. The Arab Levant countries, being amidst a political turmoil, had the inadequacies and the fragility of their public health systems exposed by the COVID-19 pandemic. The unrest in Lebanon, the uprising in Iraq, the restrictions placed on Syria, and the economic difficulties in Palestine have all played an important role on poor management of the pandemic. Jordan, on the contrary, is a good example of a strong state, able to implement proper measures without public dissent, and should serve as a model for countries of the Arab Levant. Therefore, political determinants, state capacity, and economic disparity shape epidemic dynamics in different ways [50, 51]. Political stability plays an important role in predicting a country’s ability to respond effectively to a pandemic. Other indicators such as government effectiveness and regulatory quality also help predict the citizens’ trust in the government and the likelihood of adhering to restrictions and measures imposed. It is vital to use these indices to predict a government’s capacity to respond to a pandemic, and to tailor an individualized plan for each country to best implement control measures.

## Data Availability

All data is public and freely available. Data for the number of COVID-19 cases and deaths was retrieved from the Lebanese Ministry of Health, the World Health Organization, and Our World in Data: https://ourworldindata.org/covid-cases https://ourworldindata.org/covid-deaths https://www.moph.gov.lb/maps/covid19.php https://covid19.who.int/ Data for the Government Response Stringency Index of each country was collected by the University of Oxford’s Oxford Covid-19 Government Response Tracker (OxCGRT), available at https://www.bsg.ox.ac.uk/research/research-projects/covid-19-government-response-tracker and at https://github.com/OxCGRT/covid-policy-tracker/tree/master/images/country_charts The Political Stability Index for each country was retrieved from the Global Economy website at https://www.theglobaleconomy.com/rankings/wb_political_stability/ Concerning the World Governance Indicators, data was collected by the World Bank and retrieved from https://info.worldbank.org/governance/wgi/Home/Reports Data for the 2019 Global Health Security Index was retrieved from the GHS report at https://www.ghsindex.org/wp-content/uploads/2020/04/2019-Global-Health-Security-Index.pdf

## Abbreviations

ALC: Arab levant countries
WHO: World health organization
OECD: Organization for economic co-operation and development
PPE: Personal protective equipment
